# Carcinoid syndrome: update on the pathophysiology and treatment

**DOI:** 10.6061/clinics/2018/e490s

**Published:** 2018-08-03

**Authors:** Anezka C. Rubin de Celis Ferrari, João Glasberg, Rachel P Riechelmann

**Affiliations:** I Departamento de Oncologia, Hospital Sirio Libanes, Sao Paulo, SP, BR; II Disciplina de Radiologia e Oncologia, Instituto do Cancer do Estado de Sao Paulo (ICESP), Hospital das Clinicas HCFMUSP, Faculdade de Medicina, Universidade de Sao Paulo, Sao Paulo, SP, BR; III Departamento de Oncologia, AC Camargo Cancer Center Sao Paulo, SP, BR

**Keywords:** Neuroendocrine Tumors, Carcinoid Syndrome, Neoplasm

## Abstract

Approximately 30-40% of patients with well-differentiated neuroendocrine tumors present with carcinoid syndrome, which is a paraneoplastic syndrome associated with the secretion of several humoral factors. Carcinoid syndrome significantly and negatively affects patients' quality of life; increases costs compared with the costs of nonfunctioning neuroendocrine tumors; and results in changes in patients' lifestyle, such as diet, work, physical activity and social life. For several decades, patients with neuroendocrine tumors and carcinoid syndrome have been treated with somatostatin analogues as the first-line treatment. While these agents provide significant relief from carcinoid syndrome symptoms, there is inevitable clinical progression, and new therapeutic interventions are needed. More than 40 substances have been identified as being potentially related to carcinoid syndrome; however, their individual contributions in triggering different carcinoid symptoms or complications, such as carcinoid heart disease, remain unclear. These substances include serotonin (5-HT), which appears to be the primary marker associated with the syndrome, as well as histamine, kallikrein, prostaglandins, and tachykinins.

Given the complexity involving the origin, diagnosis and management of patients with carcinoid syndrome, we have undertaken a comprehensive review to update information about the pathophysiology, diagnostic tools and treatment sequence of this syndrome, which currently comprises a multidisciplinary approach.

## INTRODUCTION

Carcinoid syndrome (CS) is a paraneoplastic syndrome associated with the secretion of several humoral factors, such as polypeptides, vasoactive amines, and prostaglandins 1. The main symptoms of CS are episodic facial flushing that may be accompanied by hypotension and tachycardia, diarrhea, bronchoconstriction, venous telangiectasia, dyspnea and ultimately fibrotic complications such as mesenteric and retroperitoneal fibroses and carcinoid heart disease (CHD) 2. CS is predominantly associated with neuroendocrine tumors (NETs) that arise from the midgut in the setting of extensive liver metastases but may be present in patients with bronchial carcinoids and, more rarely, in patients with pancreatic NETs. In patients with extensive liver metastases, a large amount of tumor-secreted substances are not completely metabolized by hepatic or pulmonary cells and enter the systemic circulation, causing carcinoid symptoms; carcinoid-produced substances may also enter the systemic circulation when the patient exhibits *forame ovale* or when the primary tumor is located in the bronchi 2.

Considering all types of NETs and all stages, among patients 65 years old or older, a large epidemiological study from the US reported that 19% of this patient population had CS 3. This study evaluated 9,512 patients with a diagnosis of NET and concluded that CS has a significant association with tumor grade, more advanced stage and midgut primary site, as well as a significant reduction in the overall survival of affected patients. CS also significantly and negatively affects patients' quality of life 4; increases costs compared with the costs of nonfunctioning NETs; and results in changes in the patients' lifestyle, such as diet, work, physical activity and social life 5. A cross-sectional study conducted in twelve countries with 1,928 patients with NETs has shown that 49% of patients miss work due to their disease 6. Among the patients who did not work, the vast majority (82%) left their work because of the illness, demonstrating the strong negative impact of NETs on patients' lives.

For several decades, patients with NETs and CS have been first treated with somatostatin analogues. While these agents provide significant relief from CS symptoms, there is inevitable clinical progression, when new therapeutic interventions are needed. Given the complexity involving the origin, diagnosis and management of patients with CS, we have undertaken a comprehensive review to update our knowledge about the pathophysiology, diagnostic tools and treatment options for this syndrome.

### Pathophysiology

**Figure 1:**
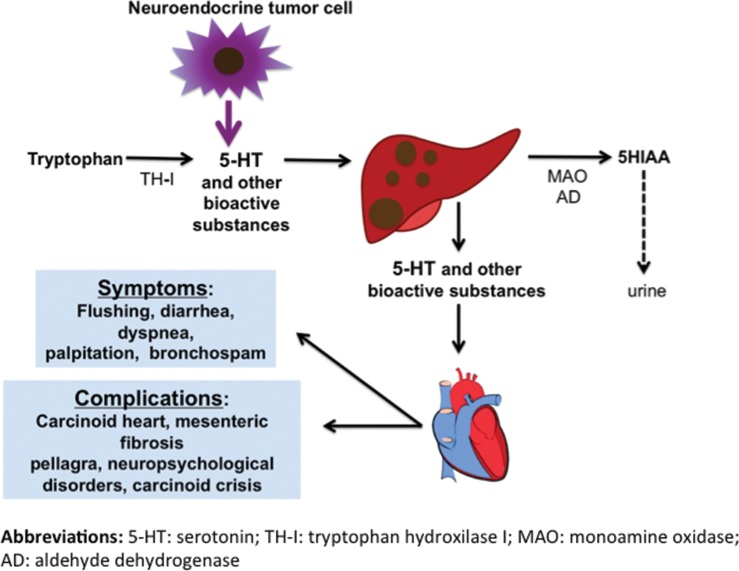
Summary of the pathophysiology of carcinoid syndrome.

More than 40 substances have been identified as being potentially related to CS; however, their individual contributions in triggering different carcinoid symptoms and complications, such as CHD, remain unclear 7. These substances include serotonin (5-HT), which appears to be the primary marker associated with the syndrome, as well as histamine, kallikrein, prostaglandins, and tachykinins 2. Below, we discuss the characteristics of the most common carcinoid symptoms and their related complications, which are summarized in Table 1 and Figure 1.

### Flushing

Flushing is a subjective sensation of warmth that is accompanied by reddening of the skin anywhere on the body, especially the face, neck, and upper torso 8. In over 90% of cases, flushing is the clinical hallmark of functional NETs and is often episodic 9. Vasoactive substances, usually secreted by functioning NETs that are located distal to the portal vein or downstream of functioning hepatocytes, such as 5-HT, substance P, histamine, catecholamines, and prostaglandins, provoke flushing when they are not inactivated by hepatocytes 10,11. The release of carcinoid-related substances is usually triggered by amine-rich foods (chocolate, banana, kiwi, avocado and nuts), alcohol and an increase in adrenergic activities, such as physical exercise. Importantly, a recent retrospective study demonstrated that the use of serotoninergic antidepressants was safe to treat depressive symptoms and did not significantly worsen carcinoid symptoms 12.

While 5-HT is the proposed main causative agent of CS-associated flushing, as evidenced by elevated levels of a known 5-HT metabolite, 5-hydroxyindoleacetic acid (5-HIAA), in the 24-h urine test, there is some speculation that other peptides are involved because some characteristics of flushing differ across patients. For example, a rapid cyanotic facial and trunk flush with a mild burning sensation that lasts less than a minute is commonly associated with midgut NETs; on the other hand, foregut tumors tend to produce pruritic wheals that are reddish-brown and occur over the entire body 9. Moreover, some NET patients present flushing with low or normal levels of 5-HIAA, while patients who have high levels of urinary 5-HIAA are totally asymptomatic 13.

### Diarrhea

The prevalence of CS-associated diarrhea among NET patients with elevated urinary 5-HIAA is as high as 60-80% 14. However, this type of diarrhea is not typical but rather unspecific, usually described as intermittent and sporadic and often accompanied by mild abdominal cramping, and it may become continuous when complicated by bacterial overgrowth 14,15. This somewhat atypical course may explain the delayed diagnosis of NETs 14, which can sometimes be after many years of uncomplicated and mild symptoms that result in many patients being diagnosed with advanced incurable disease. Because 5-HT acts physiologically by stimulating intestinal motility and intestinal secretion, high levels of 5-HT could induce increased frequency of bowel movements and decrease stool consistency; this is observed clinically as patients with CS diarrhea and is described as secretory. For example, a preclinical study 16 using human intestinal mucosa that was resected during surgery for Crohn's disease showed that 5-HT induces secretion across the human ileal mucosa via the 5-HT_4_ subtype receptors, whereas the 5-HT_2A_ receptor appears to mediate the gut effects of 5-HT in the human sigmoid colon 16. The most recent evidence that CS is caused by elevated 5-HT is the significant reduction in the frequency of daily bowel movements and urinary 5-HIAA levels demonstrated in the phase III placebo-controlled trial of telotristat ethyl, an oral inhibitor of 5-HT synthesis 17. Nevertheless, other substances may be cosecreted with 5-HT, causing diarrhea 18. For example, other physiological factors normally secreted by endocrine gut cells, such as histamine, kallikrein and substance P, could, if overproduced, lead to diarrhea 19. Methods to properly measure the serum levels of these substances are needed.

### Bronchospasm

There are very few studies on CS-associated bronchospasm. In a retrospective study with 748 CS patients, the prevalence of bronchospasm was 15% 20. The underlying mechanism is not clear. Patients who complain about bronchospasm tend to report concurrent flushing, sneezing and dyspnea. Secretion of histamine and 5-HT by the tumor are probably linked to the mechanisms of bronchospasm.

### Carcinoid crisis

A carcinoid crisis is a serious and potentially life-threatening exacerbation of CS due to the release of large amounts of amines in the circulation. Carcinoid crisis is characterized by hypotension, arrhythmias, tachycardia, flushing and bronchospasm, and it can be lethal. Carcinoid crisis can occur spontaneously, but it is more common after stressful procedures such as anesthesia, surgery or radiologic interventions. While prophylactic use of short-acting somatostatin analogues has been suggested and widely used to prevent carcinoid crisis that could be induced by invasive procedures, some patients still experience uncontrolled carcinoid symptoms 2,21. Recently, Massimino et al. 22 reported that a 500-μg bolus of octreotide acetate administered preoperatively was insufficient to prevent intraoperative carcinoid crisis. In contrast, Woltering et al. 23 observed that only 3.4% of CS patients experienced intraoperative crisis using continuous infusion of IV octreotide acetate preoperatively. Given the life-threatening consequences of carcinoid crisis, we recommend that prophylactic and intraoperative octreotide be administered to all patients with CS and/or elevated urinary 5-HIAA levels when they are treated with invasive interventions such as hepatic embolization and liver biopsy as well as during surgical procedures 24.

## SYMPTOMS ASSOCIATED WITH TRYPTOPHAN OR NIACIN DEPLETION

In a normal context, 99% of tryptophan is used in the synthesis of nicotinic acid, while the remaining is utilized to produce 5-HT. Tryptophan is catabolically degraded by two competing pathways that produce either 5-HT or beta-nicotinamide adenine dinucleotide (NAD), which is an active form of niacin (vitamin B3). Since tumors associated with CS can consume up to 60% of tryptophan in the human body 25, if not properly treated, patients may experience adverse events resulting from tryptophan and/or niacin deficiencies. Such a state of deprivation may cause several problems, including pellagra and neurocognitive disturbances, as discussed below.

### Neuropsychological symptoms

It has been estimated that extreme tryptophan deprivation can lead to an 87% to 97% reduction in the levels of 5-HT synthesized in the brain 26. Russo et al. 27 demonstrated lower tryptophan serum levels in CS patients. Pasieka et al. 28 compared 36 CS patients with 20 healthy controls through self-report cognitive questionnaires and a battery of six standardized neurocognitive tests. They demonstrated that patients with CS presented greater cognitive dysfunction than healthy controls. Supporting the concept that CS induces neurocognitive dysfunction through tryptophan depletion, studies with drugs that inhibit 5-HT synthesis have reported associated depressive symptoms. In 1967, Engelman et al. 29 tested para-chlorophenylalanine, a drug that inhibits tryptophan hydroxylase (TPH), which is the enzyme involved in 5-HT synthesis, in patients with CS. While the investigators observed reduced levels of 5-HIAA and CS symptom improvement, they described severe neurological adverse effects, including major depression, and the clinical development of this drug was halted. Recently, in the phase III placebo-controlled trial TELESTAR 30 with telotristat ethyl, patients who received 500 mg of telotristat three times per day (TID) reported more depressive symptoms (15.6% of patients taking telotristat *versus* 6.7% of patients taking the placebo) than those who were treated with 250 mg TID or placebo. Longer duration of treatment with telotristat ethyl and more studies with a larger number of patients are necessary to properly evaluate the neurological effects of this drug.

However, it is not possible to infer direct and exclusive causality between tryptophan depletion and neurological impairment given that the cancer itself may lead to emotional stress that could affect cognition; other tumor-secreted substances and malnourishment resulting from uncontrolled diarrhea could also contribute to neurological/cognitive dysfunction. Longitudinal studies with control groups of healthy and nonfunctioning NET patients could better evaluate whether and to what extent neurocognitive dysfunction occurs in patients with CS.

### Pellagra

**Figure 2:**
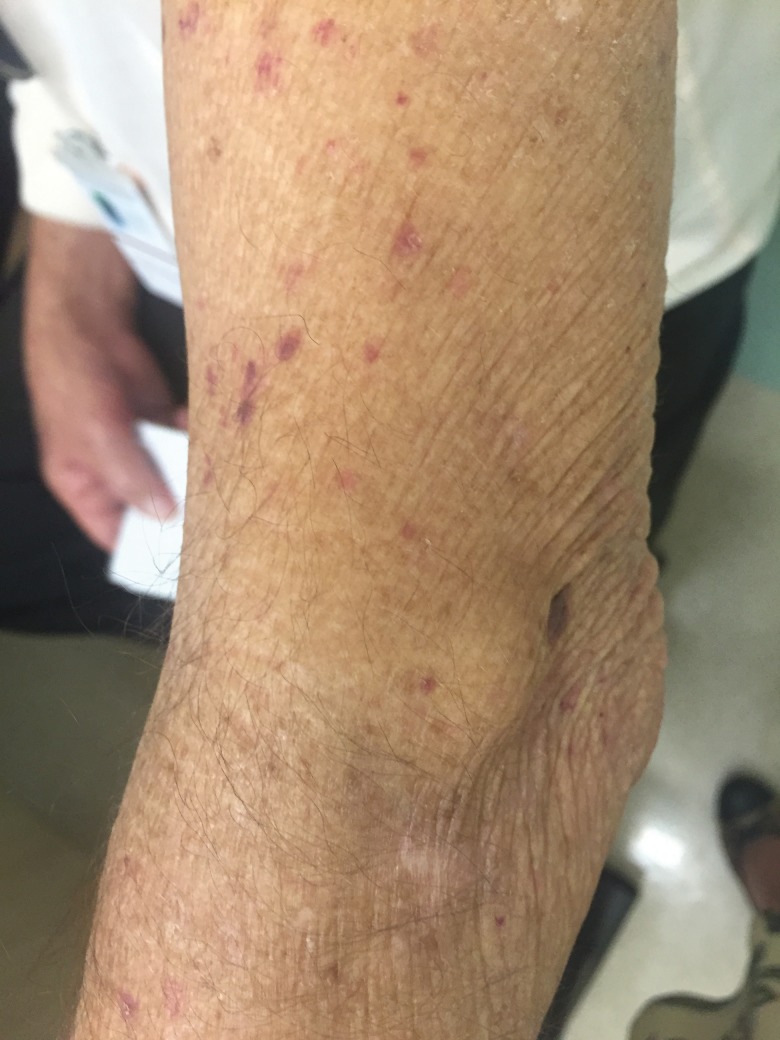
Patient with pellagra, in whom we observed dry skin and scratches from intense itching.

Pellagra is a clinical condition caused by niacin deficiency (vitamin B3) characterized by dermatitis, diarrhea and dementia in severe cases. The actual prevalence of pellagra in patients with CS is unknown, although some studies have reported that approximately 5% of CS patients experience pellagra 31. Fortunately, pellagra is rarely seen after the incorporation of somatostatin analogues into the therapeutic arsenal for NET treatment. However, in developing countries, somatostatin analogues are not available in the public systems, such as in the public health system of Brazil. In such settings, it is not uncommon to see CS patients presenting with long-term uncontrolled symptoms, including intense skin pruritus resulting from pellagra. Figure 2 demonstrates a patient with pellagra and niacin deficiency due to long-term CS that was not adequately controlled due to lack of adherence to somatostatin analogues.

## SYMPTOMS ASSOCIATED WITH FIBROTIC COMPLICATIONS

### Carcinoid heart disease

CHD is part of the CS spectrum and is associated with high mortality and morbidity 2. Its incidence varies, ranging from 19% to 60% 1, depending on the patient population, access to somatostatin analogues, definition of CHD and methods used for diagnosis 1,2. CHD is characterized by deposition of fibrous tissues on the valves primarily affecting the right heart chambers, particularly the tricuspid valve, because circulating 5-HT is inactivated in the lungs 32. Therefore, left valve involvement is rare and is usually associated with a higher risk of interatrial shunt that may be present in these patients due to overload of the right atrium.

The exact mechanism underlying the development of CHD remains unclear. However, there is strong evidence for the role of 5-HT in the fibrogenesis and stimulation of fibroblast growth in CHD 2. Several studies have shown that urinary levels of 5-HIAA were significantly higher in patients with CHD than in those without this condition 1,33. There are seven classes of 5-HT receptors, and 5-HT_2B_ is the receptor in the cardiovascular system that may be involved in fibrogenesis 34. The following evidence points to an important role of 5-HT in the development of CHD: serotonergic drugs (fenfluramine, pergolide, cabergoline, and ergotamine by-products) have been shown to cause valve fibrosis similar to that observed with CHD 35; *in vitro* studies have shown the mitogenic action of 5-HT in fibroblasts, smooth muscle cells, and endothelial cells 36,37; studies with animals exposed to high levels of IV 5-HT showed that they developed cardiac valve dysfunction 38-40; and lastly, it has been demonstrated in animal models that hyperexpression of 5-HT_2B_ receptors in the heart leads to cardiac hypertrophy because of increased deposition and remodeling of the extracellular matrix 41, whereas deletion of 5-HT_2B_ leads to ventricular dilation and incomplete development of the heart 42. Therefore, activation of the 5-HT_2B_ receptor triggers distinct intracellular signaling pathways, which in turn may result in a stronger inflammatory response and release of cytokines including TNF-alpha 43, activation of the MAPK 44 signaling pathway and hyperexpression of TGF-beta 45, all converging to cardiac fibrosis.

Despite the strong evidence of 5-HT involvement in the development of CHD, a significant proportion of patients with elevated plasma levels of 5-HT do not develop this condition 46, which suggests that there are other biochemical or genetic mechanisms involved. For example, other potential contributing factors are bradykinins, tachykinins, activin A and tissue growth factor (CTGF). Bradykinins 46 are associated with endocardium injury that results in fibrosis as a response mechanism of the endocardium 46. In addition, tachykinins have been described as endocardial fibroblast pro-proliferative agents that induce plaque formation. Activin A is a cytokine and a member of the TGF-beta superfamily with fibrogenic properties, and its expression has been demonstrated in fibrotic plaques of patients with CHD 47. Hyperexpression of CTGF and TGF-beta1 mRNA in intestinal NETs, which together promote the overproduction of collagen and fibrosis, has been described by Kidd et al. 48,49; the overexpression of TGF-beta1 50 is also stimulated by 5-HT_2B_ receptors, mostly found in the heart.

Beyond tumor-related risk factors for CHD, clinical features may be associated with the onset of CHD among CS patients. In a case-control study of 42 NET patients with or without CHD but all with elevated urinary 5-HIAA levels that was conducted by our group, we found that 38% (95% confidence interval: 23 to 54%) presented with CHD, defined as at least moderate tricuspid valve regurgitation 51. In our study, a concurrent or prior diagnosis of cardiovascular comorbidities was associated with CHD [odds ratio 6.58 (1.09; 39.78), *p*=0.040]. Patients with cardiovascular diseases often present endothelial dysfunction, marked by chronic endothelial inflammation and abnormalities in platelet aggregation function, and it is possible that CHD patients have a higher concentration of 5-HT in platelets in the heart, leading to cardiac fibrosis through activation of 5-HT_2B_ receptors 52.

While the initial disease of CHD is usually asymptomatic, symptoms of right heart failure, such as peripheral edema, abdominal discomfort and indigestion, early satiety and cachexia, appear during the course of its evolution. As the condition worsens, fatigue, dyspnea upon exertion, jugular swelling, and ascites are observed. If these symptoms are not treated, progression of the manifestations leads to death from heart failure.

### Mesenteric fibrosis

Mesenteric fibrosis (MF) is another complication of uncontrolled CS. At least some signs of MF occur in approximately 50% of CS patients 53, g consequent to a fibrotic and desmoplastic reaction around metastatic mesenteric lymph nodes. MF is a pathognomonic radiological sign of midgut NET, which can be observed on computerized tomography and nuclear magnetic resonance images 54-56. MF is usually detected radiologically and may have the appearance of a mesenteric mass with the opacity of soft tissue radiating outward in a “wheel spoke” appearance 53. MF can lead to ischemia of vessels and intestinal obstruction. This vascular ischemia can lead to bowel congestion and result in decreased absorption of nutrients and can also cause ascites and more severe cases of mesenteric ischemia. Another rare complication of MF is ureteral obstruction, which may cause renal failure 54.

5-HT secreted by NETs appears to play a central role in MF. A recent study demonstrated a significant association of MF with elevated urinary 5-HIAA levels, suggesting a potential causal relationship 54. Another study with 52 patients with midgut NETs demonstrated that elevated platelet 5-HT is associated with the presence of a mesenteric mass 57.

Another substance that may be involved in pathophysiology of MF is TGF-beta, which may play a critical role in fibrogenesis due to its known ability to stimulate collagen synthesis 58.

### Diagnosis

The diagnosis of CS requires the combination of carcinoid symptoms and evidence of elevated levels of 5-HIAA in a 24-h urine sample. The sensitivity and specificity of this test for CS are higher than 90% in patients with characteristic symptoms 59. However, clinicians should be aware of false positive findings that could be due to the use of serotoninergic drugs (antidepressants, tramadol, acetaminophen, salicylates, L-DOPA) and patients' consumption of foods rich in 5-HT. Therefore, to increase the accuracy of the diagnosis, it is advisable to avoid using serotonergic drugs and avoid foods such as banana, kiwi, avocado, pineapple, nuts, coffee, etc., for 2 days before and during the 24-h urine collection.

The diagnosis of CHD is performed by transthoracic echocardiography, which shows valve thickening with retraction and reduction in the mobility of the tricuspid valve in patients with CS 2,24. Nuclear magnetic resonance imaging of the heart also allows analysis of ventricular anatomy and function, thereby contributing to a better assessment of valvular changes when the echocardiogram is not conclusive 32. NT-proBNP, a marker of heart failure, is a useful serum biomarker in the screening of heart disease in patients with CS.

**Figure 3:**
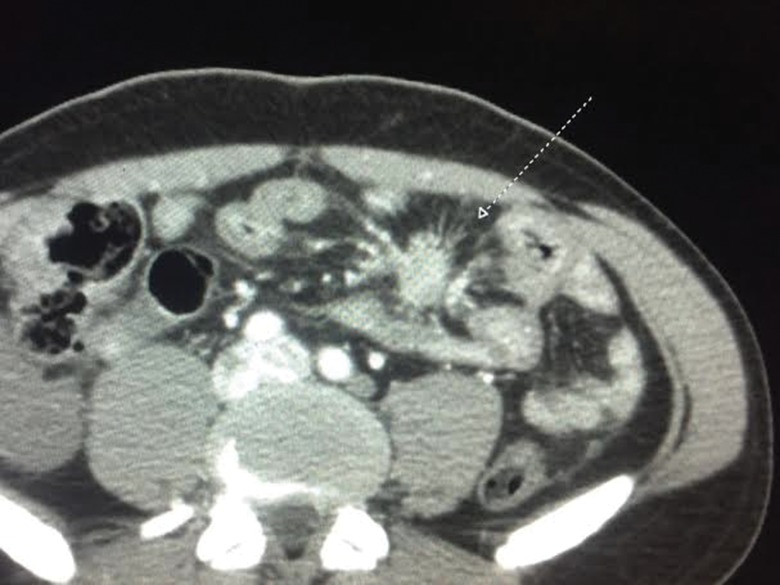
CT showing a characteristic image of mesenteric fibrosis.

MF is diagnosed with an abdominal imaging exam (usually tomography or NMR) that shows at least a spiculated mass with attenuation in the soft tissue range, with fibrotic bands that radiate outward from the mesenteric fat tissue with a star pattern around a metastatic lymph node 56. Figure 3 shows a typical case of MF secondary to CS from midgut NET.

### Treatment

The main pillar of treatment for CS is the use of somatostatin analogues, such as octreotide and lanreotide. Approximately 80% of well-differentiated tumors express somatostatin receptors in the NET cell surface 60. Octreotide and lanreotide bind to somatostatin receptors and inhibit the secretion of several hormones and vasoactive substances, thus improving flushing and diarrhea symptoms in over 80% patients with CS 61. Octreotide in its depot form is called Octreotide LAR and can be administered intramuscularly at a dose of 20 to 30 mg every 4 weeks. Lanreotide is administered subcutaneously at a dose of 120 mg every 4 weeks. The Brazilian Consensus of NETs considers octreotide and lanreotide to be similarly safe and effective in the antitumor and symptomatic treatment of well-differentiated gastroenteropancreatic NETs 24. In addition, phase III placebo-controlled trials 62,63 have also demonstrated that these agents provide antiproliferative effects, prolonging progression-free survival.

Unfortunately, all patients experience symptomatic progression of CS after a median of several months to years. In this scenario, several therapeutic options have been successfully tested and are discussed below.

## REFRACTORY CARCINOID SYNDROME

There is no uniform definition of refractory CS, which makes it difficult to compare results from different studies. Most studies consider refractory CS as uncontrolled carcinoid symptoms despite label doses of somatostatin analogues 64. However, “uncontrolled” is a subjective term. Except for two randomized trials, the TELESTAR 17 and the Passport 65, the overwhelming majority of studies proposing treatments for refractory CS are either retrospective or subgroup analyses of randomized trials. However, despite the low level of evidence for many therapeutic options to treat refractory CS, there is consistency in the clinically significant results for most of them.

Some patients with difficult-to-control diarrhea and flushing may benefit from more frequent doses of octreotide or lanreotide 66. Some problems may also occur with the administration of somatostatin analogues, such as decreased absorption and tachyphylaxis. Related problems due to decreased absorption by fibrosis from the injection site, for example, can be avoided through better training of the nursing staff. However, tachyphylaxis, which causes a shorter duration of the medication effect, with symptom control for 2 to 3 weeks only, can be reduced by shortening the interval of drug application, such as every 21 days 67. If disease control is not achieved after these strategies, it is still possible to switch to a somatostatin analogue. A phase II study and case reports have shown short-term improvement in symptoms after switching from lanreotide to octreotide or otherwise, which can be explained by differences in the receptor affinity of drugs 68.

Telotristat ethyl 17,30 was recently approved by the US Food and Drug Administration. Together with octreotide, telotristat reduces the frequency of diarrhea. A prospective placebo-controlled trial 30 demonstrated that telotristat significantly reduced diarrhea and urinary levels of 5-HIAA. The effect of telotristat on facial flushing and CHD needs to be clarified in further studies. Unfortunately, the Passport trial, which tested pasireotide for the treatment of refractory CS, was ineffective in significantly controlling CS when compared with octreotide LAR 60 mg 65.

An alternative to somatostatin analogues for the treatment of refractory diarrhea and flushing is interferon alpha, which, in retrospective studies, has been shown to provide symptom relief in up to 40-50% patients 69. In addition, the use of interferon alpha may be associated with some antitumor effects by stimulating T lymphocytes 70; however, objective responses are rare 71, and the toxicity profile is unfavorable.

Retrospective studies suggest symptomatic benefit with the use of everolimus in patients with CS 72. Additionally, the phase III placebo-controlled trial RADIANT 2 73 demonstrated that everolimus combined with octreotide led to more reduction in the levels of urinary 5-HIAA in comparison with the levels achieved with octreotide only, although information about symptomatic response was lacking.

Local liver therapies may be considered for patients with liver metastases, with the aim of managing the symptoms of CS and controlling the disease. In the case of potentially resectable lesions, hepatectomy 74 may be considered, with long-term disease-free survival in up to 20% patients with complete resection. When liver lesions are not resectable, the indication of cytoreductive surgery is more controversial, but some retrospective studies 75-79 suggest improved palliation of symptoms. Another effective therapy for refractory CS is liver transarterial embolization, which can control carcinoid symptoms in up to 75% of patients 80-82. Importantly, short-acting octreotide should be administered before, during and after the procedure in all these situations to avoid a carcinoid crisis 24.

Another promising strategy is the use of somatostatin analogue radiolabeled peptide therapy (PRRT) with lutetium^177^ or yttrium^90^ to provide radiation directed to the cells that express somatostatin receptors. Studies suggest significant symptom improvement with PRRT 83,84. Prior to PRRT, it is mandatory to quantify cells with somatostatin receptors using imaging exams before treatment (octreoscan or PET-Ga68 DOTATATE). A similar strategy has been used with meta-iodobenzylguanidine (MIBG), a biogenic amine that is captured by cells of neural origin and radiolabeled with iodine. Pathirana et al. 85 showed a positive I-123 MIBG scan test for more than 60% of the patients with well-differentiated midgut NETs. Small studies suggest symptom control for refractory patients 85,86.

NETs with CS usually respond poorly to conventional cytotoxic chemotherapy, and this type of approach is not recommended 24,87.

The optimal treatment sequence to obtain the best control of the disease remains unknown. A recent systematic review conducted by our group 64 proposed a therapeutic sequence to manage refractory CS, taking into account the type of progression: only symptomatic or also radiological.

## CARCINOID HEART DISEASE

The treatment of CHD includes the management of volemia and heart failure, treatment of the NET itself, reduction in the production of related hormones (with the use of somatostatin analogues), and heart valve repair surgery. The use of somatostatin analogues, such as octreotide and lanreotide, improves CS symptoms and reduces urinary levels of 5-HIAA. However, studies suggest that their use does not lead to regression of CS or prevent its occurrence 88. Bosentan, an oral antagonist of the endothelin receptor, has been tested in a small trial of six patients with CHD and New York Heart Association (NYHA) functional class III or more, demonstrating that the right ventricular systolic pressure decreased after 3 and 6 months; the 6-minute walk distance also significantly improved, and five out of six patients improved their NYHA functional class 89. More studies are needed to replicate these encouraging results with bosentan.

Because telotristat ethyl reduces the levels of 5-HIAA in 24-h urine tests, it is may be a promising treatment to prevent or delay the onset of CHD.

Until now, the treatment that best controls CHD is heart valve repair surgery; however, it is associated with a high rate of mortality (perioperative mortality is between 10% and 20% in highly specialized centers) 2. Repair surgery is indicated only for symptomatic patients who are refractory to clinical treatment or who have right ventricular dysfunction 90. However, these are the patients with a higher risk for unfavorable outcomes.

As for MF, once present, there is no specific treatment to alleviate the symptoms associated with this CS complication. However, its development can be prevented by resection of the primary midgut 5-HT-producing NET, and this is recommended by guidelines 24,87.

CS secondary to well-differentiated NETS has a heterogeneous clinical presentation ranging from frustrating symptoms such as mild diarrhea and flushing, often misdiagnosed with menopause symptoms, to symptoms that significantly worsen the patient's quality of life, such as difficult-to-control diarrhea and fibrotic complications such as MF and CHD. While it is known that 5-HT plays a central role in this entity, it is insufficient to justify all the symptoms, and the pathophysiology of CS is not yet fully understood.

Somatostatin analogues (octreotide and lanreotide) are the standard treatment for CS and have excellent efficacy. Unfortunately, the disease progresses, and patients experience carcinoid symptoms that are refractory to label doses to somatostatin analogue treatment. In this scenario, locoregional therapies and systemic treatments can be helpful. Telotristat ethyl, a new drug that inhibits 5-HT synthesis, appears to be a promising drug for the control of refractory CS.

However, there are many more questions than answers regarding CS. Therefore, we strongly advocate collaborative efforts among researchers, institutions and funding agencies to develop preclinical research to investigate the mechanism underlying CS, to conduct clinical trials with patients suffering from CS, to study the prognostic and predictive factors of current treatments and to generate epidemiological data to evaluate the real impact of CS.

## AUTHOR CONTRIBUTIONS

Ferrari AC wrote 65% of the manuscript and reviewed and corrected it. Glasberg J wrote 20% of the manuscript. Riechelmann R wrote 15% of the manuscript, guided the article and reviewed it.

## Figures and Tables

**Table 1:** Symptoms and signs of carcinoid syndrome.

Symptom	Frequency %	Characteristics	Involved mediators
Flushing	90 9	Foregut: long-lasting, purple 9-11	Catecholamines, 5-HT, histamine, substance P 10,11
		Midgut: short-lasting, pink to red color, face and trunk, can occur several times a day for a few minutes 9-11	Catecholamines, histamine, substance P, prostaglandins 10,11
Diarrhea	60-80 14	Secretory: intermittent accompanied by abdominal cramping 14,15	Gastrin, 5-HT, histamine, prostaglandins, VIP 16,19
Abdominal pain	35	Progressive 54	Small bowel obstruction, hepatomegaly, ischemia 2
Bronchospasm	15 20	Wheezing 20	Histamine, 5-HT 20
Pellagra	5 31	Dermatitis, diarrhea, dementia 31	Niacin deficiency
CHD	19-60 32,33	Dyspnea, holosystolic murmur radiating to the right side of the chest 32	5-HT, bradykinins, tachykinins, activin A, tissue growth factor 32
Mesenteric Fibrosis	50 53	Ischemia of vessels: decreased absorption of nutrients, ascites, intestinal obstruction, ureteral obstruction 54	5-HT, TGF-beta 53,58
